# Serum levels and gene polymorphisms of angiopoietin 2 in systemic lupus erythematosus patients

**DOI:** 10.1038/s41598-020-79544-z

**Published:** 2021-01-08

**Authors:** Jia-Min Wang, Wang-Dong Xu, Zhi-Chao Yuan, Qian Wu, Jie Zhou, An-Fang Huang

**Affiliations:** 1 Department of Evidence-Based Medicine, School of Public Health, Southwest Medical University, 1 Xianglin Road, Luzhou, 646000 Sichuan People’s Republic of China; 2 Department of Rheumatology and Immunology, Affiliated Hospital of Southwest Medical University, 25 Taiping Road, Luzhou, 646000 Sichuan People’s Republic of China

**Keywords:** Genetics, Diseases, Rheumatology

## Abstract

This study aimed to discuss association between serum Angiopoietin2 (Ang2) levels, *Ang2* gene polymorphisms and systemic lupus erythematosus (SLE) susceptibility. It was carried out by 235 SLE, 342 other inflammatory autoimmune diseases patients and 380 healthy individuals. Serum Ang2 levels was examinated by ELISA, and Ang2 rs12674822, rs1823375, rs1868554, rs2442598, rs3739390 and rs734701 polymorphisms were genotyped using KASP. Increased Ang2 concentrations in SLE patients were observed compared with healthy controls and patients with other inflammatory autoimmune diseases. For allelic contrast, except for rs1823375 (P = 0.058) and rs2442598 (P = 0.523), frequencies of alleles for other polymorphisms were significantly different between SLE patients and controls. Genotypes for rs12674822 (TT), rs1868554 (TT, TA and TT+TA), rs734701 (TT) were negatively correlated with SLE susceptibility (OR = 0.564 for rs12674822; OR = 0.572, OR = 0.625, OR = 0.607 for rs1868554; OR = 0.580 for rs734701). Patients carrying rs1868554 T allele and rs3739390 G allele were more likely to develop hematuria (P = 0.039; P = 0.003). The G allele frequencies of rs12674822 and rs2442598 were higher in SLE patients with proteinuria (P = 0.043; P = 0.043). GC genotype frequency of rs3739390 was higher in patients with ds-DNA (+) (P = 0.024). In summary, SLE had increased serum Ang2, which may be a potential biomarker, and the polymorphisms correlated with SLE.

## Introduction

Systemic lupus erythematosus (SLE) is a kind of complicated autoimmune disease. One of the diverse clinical manifestations is vasculitis caused by endothelial cell injury^1^. Prevalence of vasculitis in SLE ranges from 11 to 36%^2^. Since inflammation exists in multiple blood vessels, the vascular damage in SLE may accompany by a variety of typical lesions, including skin vasculitis, glomerulonephritis and cerebrovascular damage^3^. To date, the specific etiology and pathogenesis of SLE have not been fully recognized. Studies showed that environment and genetics play key roles in SLE. Large-scale genetic association researches have demonstrated the significant impact of genetic susceptibility on development of SLE by identifying about 100 SLE genetic loci^4^.

Angiogenesis is potential in development of chronic inflammatory diseases and mediates acute inflammation to chronic inflammation^5^. Angiopoietin (Ang) is closely related to angiogenesis. The Ang family consists of four ligands, Ang1, Ang2, Ang3 and Ang4. Ang1 and Ang2 are involved in angiogenesis. Ang1 stabilizes and promotes maturation of unstable vessels via activating tyrosine kinase receptor 2 (Tie2) receptors. Conversely, Ang2 is originally described as a Tie2 natural antagonist, which can contribute to vascular instability, including vascular leakage, abnormal vascular structure^1,6,7^. Recent studies showed that Ang1 and Ang2 had anti-inflammatory and pro-inflammatory effects, respectively^8^. Increased levels of serum Ang2 were observed in both newly diagnosed and interferon-β treated multiple sclerosis patients^9^. In atopic dermatitis patients, higher serum Ang2 levels were detected when compared to controls, and this increase was more significant in patients with severe disease activity^10^. Ang2 expression in synovial membrane of collagen-induced arthritis mice was increased as compared to that in wild-type mice^6^. These findings indicated that Ang2 may be abnormally expressed in inflammatory autoimmune diseases.

Ang2 is a 75 kDa molecule mainly secreted by endothelial cells. The gene encoding Ang2 is located on chromosomes 8p23.1^11^. Previous studies have shown that Ang2 gene polymorphisms associated with risk of several autoimmune disorders. Rheumatoid arthritis (RA) patients in Chinese origin carrying TT allele of Ang2 rs2442598 polymorphism had a higher risk of this disease compared with those with AA genotype. Genotypes of rs1823375 and rs12674822 polymorphisms were related to the development of RA. With respect to psoriasis vulgaris (PV) patients, rs2442598 polymorphism of Ang2 related to susceptibility of the disease^12^. Interestingly, there was a higher level of serum erythrocyte sedimentation rate in RA patients carrying T allele of rs734701^13^. To date, the relationship between *Ang2* gene polymorphisms and susceptibility of SLE has not been elucidated and concentration of Ang2 in lupus was limitedly discussed. Therefore, in present study, we detected serum concentration of Ang2 in SLE patients by a large sample size, assessing if serum Ang2 could be a biomarker of SLE. In addition, we discussed the correlation between rs12674822, rs1823375, rs1868554, rs2442598, rs3739390 and rs734701 polymorphisms of *Ang2* gene and the risk of SLE.

## Methods

### Subjects

A total of 235 patients diagnosed with SLE according to the 1997 American College of Rheumatology (ACR) criteria for SLE^14^ were recruited from the Department of Rheumatology and Immunology, Affiliated Hospital of Southwest Medical University and Minda Hospital of Hubei Minzu University. Three hundred and eighty volunteers without autoimmune diseases were selected as healthy controls. This study has two parts. The first one is to determine whether serum Ang2 can distinguish SLE patients from healthy individuals, other patients with autoimmune diseases. A training cohort comprised of 58 SLE patients and 95 healthy individuals was conducted to evaluate difference of serum Ang2 levels. Then, a validation cohort with 439 patients confirmed the potential of serum Ang2 as a marker for SLE, including 97 patients with SLE, 90 with RA (conforming to 1987 ACR criteria for RA^15^), 90 with osteoarthritis (OA) (1986 ACR criteria for OA^16^), 90 with gout (2015 ACR for gout^17^), 37 with Sjogren's syndrome (SS) (2016 ACR for SS^18^) and 35 with ankylosing spondylitis (AS) (Modified New York criteria for AS^19^). SLE disease activity index (SLEDAI) evaluated disease activity of lupus patients (less active disease activity: SLEDAI < 10; active disease activity: SLEDAI ≥ 10). The second part of this study is to discuss association between *Ang2* gene polymorphisms and SLE risk, where we detected *Ang2* gene polymorphisms in 235 SLE patients and 380 healthy controls in a Chinese Han population. All participants signed informed consent before enrollment and then blood samples were collected. The study was approved by Ethics Committee of Southwest Medical University and Hubei Minzu University. We confirmed that all methods were carried out in accordance with relevant guidelines and regulations in these institutions. Clinical and demographical characteristics of patients with SLE and healthy controls are summarized in Table 1.

**Table 1:** Characteristics of SLE patients and controls.

Characteristics	SLE	HC	*P* value
Age (years)	37.72 ± 12.78	39.31 ± 9.94	0.079
Female (%)/male (%)	90.64/9.63	91.58/8.42	0.689
Arthritis, n (%)	101 (42.98)	–	–
Rash, n (%)	96 (40.98)	–	–
Alopecia, n (%)	57 (24.26)	–	–
Fever, n (%)	42 (17.87)	–	–
Hypocomplementemia, n (%)	123 (52.34)	–	–
ds-DNA ( +), n (%)	57 (24.26)	–	–
Thrombocytopenia, n (%)	31 (13.19)	–	–
Hematuria, n (%)	80 (34.04)	–	–
Proteinuria, n (%)	108 (45.96)	–	–

SLE, systemic lupus erythematosus; HC, healthy controls.

### Ang2 levels quantification

Venous blood was collected following an overnight fast. Serum was obtained by centrifugation and stored at -− 80 °C until use. Concentrations of Ang2 were detected by specific enzyme-linked immunosorbent assay (ELISA) (Cusabio, Houston, USA). All experiments were carried out in accordance with the instructions. In brief, serum samples were added into wells, and incubated at 37 °C for 2 h. After removing the liquid, biotin-antibody was added, and then incubated for 1 h at 37 °C. Subsequently, avidin conjugated horseradish peroxidae (HRP), 3,3′,5,5′-tetramethylbenzidine (TMB) substrate and stop solution were added successively and incubated for a suitable time, respectively. Each sample was detected in duplicate. The data was measured at 450 nm. The lowest detectable level of Ang2 is 9.75 pg/ml.

### Genotyping analysis

Genomic DNA was extracted from the peripheral blood using TIANamp Blood DNA kits (TIANGEN, Beijing, China), and then stored at − 80 °C. Ang2 rs12674822, rs1823375, rs1868554, rs2442598, rs3739390 and rs734701 polymorphisms were genotyped using KASP (Gene Company, Shanghai, China). Information of KASP primers (Primer_AlleleFAM, Primer_AlleleHEX and Primer_Common) was listed in Supplementary Table 1.

### Statistical analysis

All data were analyzed by GraphPad Prism version 5.01 (GraphPad Software, San Diego CA, https://www.graphpad.com/scientific-software/prism/) and the Statistical Package for the Social Science version 17.0 (SPSS Inc, Chicago, IL, https://www.ibm.com/products/spss-statistics). Quantitative data conforming to normality was shown as mean ± standard deviation (SD), otherwise, median (range) was used. Categorical data were expressed as frequency (percentage). Comparison of quantitative values was conducted by Student's t-test or Mann–Whitney U test. Categorical data were performed by Chi-square test. Ability of serum Ang2 as a marker for lupus was assessed by area under the curve (AUC) of the receiver operating characteristic (ROC) curve. Distribution of genotypes and alleles in patients and healthy controls was evaluated by Chi-square test or Fisher's exact test. Odds ratio (OR) and its 95% confidence interval (95% CI) were calculated by logistic regression model. The difference of serum levels of Ang2 between different genotypes was determined by the Kruskal–Wallis test. P < 0.05 was statistically significant.

## Results

### Serum levels of Ang2 in SLE patients in training cohort

**Figure 1:**
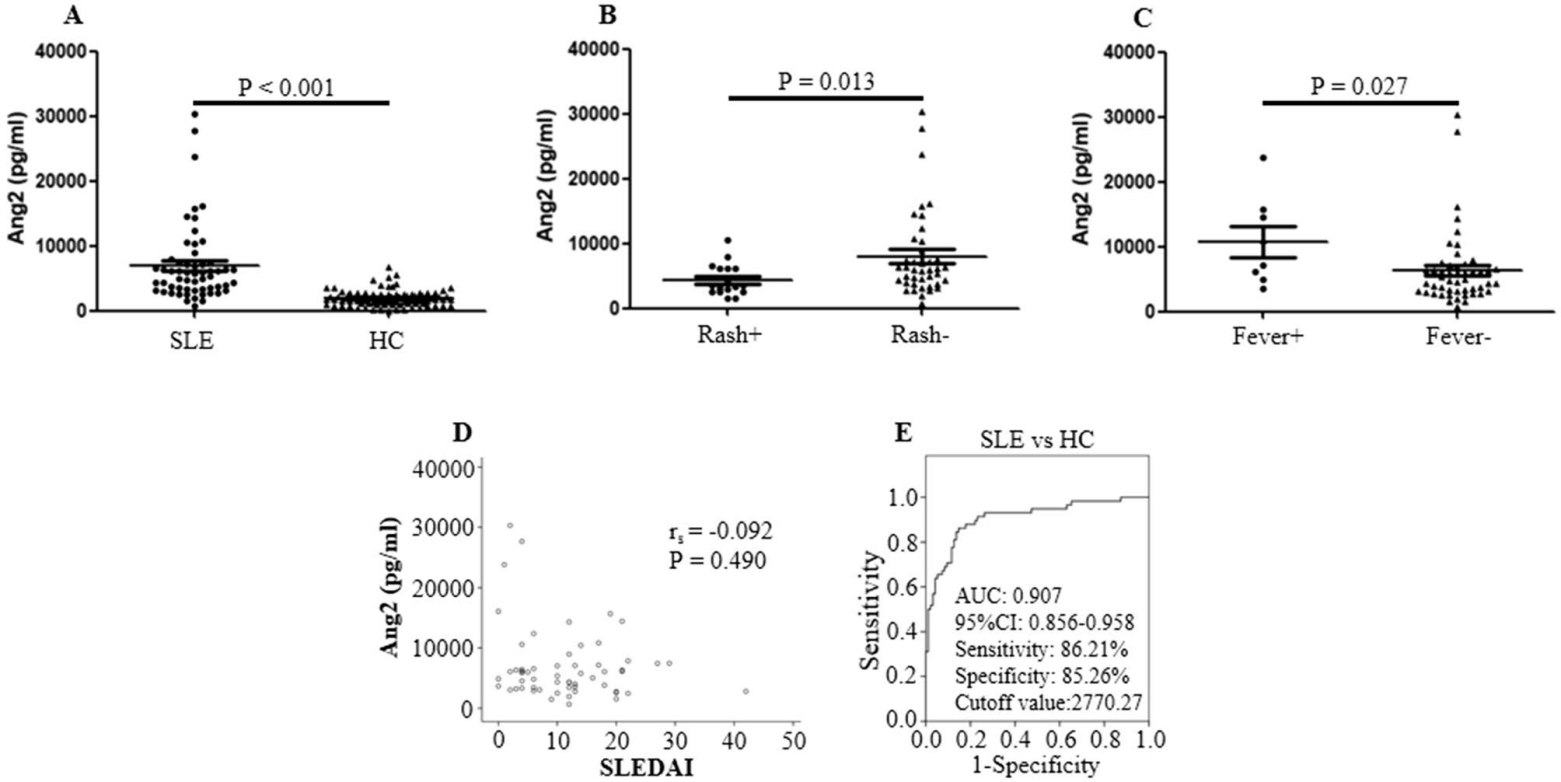
Comparison of Ang2 concentrations between SLE patients and healthy controls in the training cohort. (**A**) Serum Ang2 levels in 58 SLE patients and 95 healthy individuals were detected by enzyme-linked immunosorbent assay. Each symbol represents an independent SLE patient and healthy control. (**B**–**C**) Ang2 expression in SLE patients distributed in accordance with rash and fever. (**D**) Correlation between SLEDAI and Ang2 levels. (**E**) Receiver-operating characteristic (ROC) curve analysis of serum Ang2 for the diagnosis of SLE. The ROC for Ang2 supplied area under the ROC curve (AUC) with its associated 95% confidence intervals, sensitivity, specificity and cutoff value. Mann–Whitney U test and associated P values are indicated. The correlation was evaluated with Spearman's nonparametric test. SLE, systemic lupus erythematosus; HC, healthy control; SLEDAI, systemic lupus erythematosus disease activity index; AUC, area under the ROC curve. The ROC curves plot (1-Specificity) % on the x-axis versus the sensitivity (%) on the y-axis.

Serum concentrations of Ang2 in 58 SLE patients were significantly higher than those in 95 healthy controls (P < 0.001, Fig. 1A). Correlation analysis showed no correlation between serum levels of Ang2 and SLEDAI score (r_s_ = − 0.092, P = 0.490, Fig. 1D). Significant differences for serum Ang2 were found between SLE patients with active disease activity (n = 34) and those with less active disease activity (n = 24) (P = 0.449, data not shown). Subgroup analysis showed that serum Ang2 concentrations in SLE patients with rash and fever were strongly different from those without these clinical symptoms (P = 0.013; P = 0.027, Fig. 1B–C). There was no significant difference in serum levels of Ang2 between patients with ds-DNA (+), proteinuria and those without these clinical manifestations (P = 0.055; P = 0.323, data not shown).

To assess the potential of Ang2 as a diagnostic marker for SLE, serum Ang2 levels were analyzed by ROC curve. Results showed that serum Ang2 had a high ability to differentiate SLE patients from healthy individuals, by which the AUC, sensitivity and specificity for SLE patients were 0.907 (0.856–0.958), 86.21% and 85.26%, respectively, at the cutoff value of 2770.27 pg/ml (Fig. 1E).

### Ang2 levels in SLE patients from validation cohort

**Figure 2:**
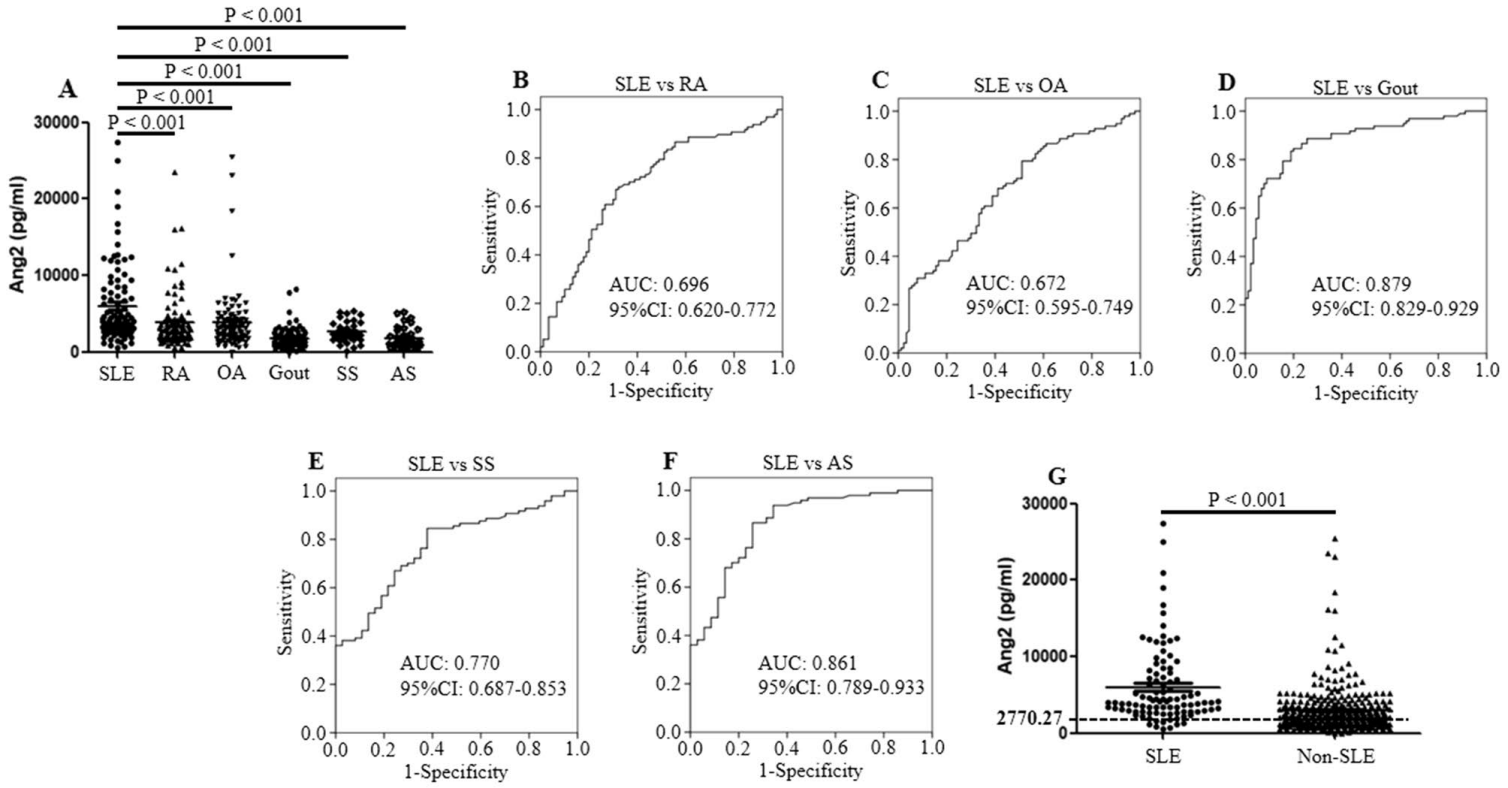
Ang2 expression in SLE patients from validation cohort. (**A**) Serum Ang2 levels in the validation cohort. Serum Ang2 protein concentrations were compared between patients with SLE (n = 97) and those with other inflammatory autoimmune diseases (n = 342, including 90 RA, 90 OA, 90 gout, 37 SS, 35 AS patients). The Ang2 levels were examined by enzyme-linked immunosorbent assay. (**B**–**F**) Receiver operating characteristic (ROC) analysis was performed to evaluate the potential of Ang2 to differentiate SLE from other inflammatory autoimmune diseases. (**G**) Analysis of the difference in serum Ang2 between SLE and non-SLE patients. The black horizontal dotted line represents the cutoff value of 2770.27 pg/ml. SLE, systemic lupus erythematosus; RA, rheumatoid arthritis; OA, osteoarthritis; SS, Sjogren's syndrome; AS, ankylosing spondylitis.

In validation cohort, the serum levels of Ang2 in 97 SLE patients were significantly higher compared with 90 RA, 90 OA, 90 gout, 37 SS and 35 AS patients (all P < 0.001, Fig. 2A). ROC analysis revealed that AUC for SLE patients was 0.696 when compared to RA (Fig. 2B). Similarly, the AUC for SLE patients was 0.672, 0.879, 0.770 and 0.861, as compared with that of OA, gout, SS and AS patients (Fig. 2C–F).

We verified the ability of serum Ang2 threshold of 2770.27 pg/ml to distinguish SLE patients from non-SLE in order to validate its potential as a disease marker for SLE. A total of 439 patients were enrolled in validation cohort, among which 201 patients had a higher concentration of Ang2 than 2770.27 pg/ml (Fig. 2G). Among the cases, 75 were diagnosed as SLE according to ACR criteria. The sensitivity and specificity were 0.77 and 0.632, respectively. The values of positive predictive and negative predictive were 0.37 and 0.91. Positive likelihood ratio (+LR), and negative likelihood ratio (-LR) were 2.09 and 0.36.

### Association of Ang2 SNPs with SLE

Hardy–Weinberg equilibrium (HWE) test in both SLE cases and healthy controls were performed. Result showed that the p value in rs12674822 of healthy controls were lower than 0.05 (P = 0.011) (Table 2). Allelic contrast and genotypes comparison were analyzed for six SNPs between SLE cases and healthy controls. For allelic contrast, except for rs1823375 and rs2442598 (G vs C: OR = 0.790, 95%CI: 0.619–1.008, P = 0.058; G vs A: OR = 0.940, 95%CI: 0.751–1.176, P = 0.586), frequencies of other alleles were significantly different between SLE patients and healthy individuals (Table 3). For rs12674822 and rs734701, frequencies of genotype TT were both significantly lower in SLE patients compared with healthy controls (TT vs GG: OR = 0.564, 95%CI: 0.358–0.890, P = 0.014; TT vs CC: OR = 0.580, 95%CI: 0.366–0.991, P = 0.020). With respect to rs1823375, frequency of GG was strongly lower in SLE patients than that in control group (GG vs CC: OR = 0.572, 95%CI: 0.339–0.964, P = 0.036). There were significant differences in genotypes distribution of rs1868554 polymorphism between SLE cases and controls (TT vs AA: OR = 0.572, 95%CI: 0.364–0.898, P = 0.015; TA vs AA: OR = 0.625, 95%CI: 0.430–0.908, P = 0.014; TT+TA vs AA: OR = 0.607, 95%CI: 0.429–0.858, P = 0.005). For rs3739390, frequencies of GC, GG+GC were lower in SLE as compared to healthy subjects ( GC vs CC: OR = 0.566, 95%CI: 0.367–0.873, P = 0.010; GG + GC vs CC: OR = 0.539, 95%CI: 0.360–0.808, P = 0.003). Genotype frequencies for Ang2 rs2442598 polymorphism were comparable between SLE cases and controls (Table 3).

**Table 2:** The Hardy–Weinberg's expectation test in SLE patients and healthy controls of six SNPs.

SNPs	SLE		HC	
	χ^2^	*P* value	χ^2^	*P* value
rs12674822	0.361	0.835	8.932	0.011
rs1823375	0.045	0.978	3.011	0.222
rs1868554	1.784	0.41	0.223	0.894
rs2442598	2.19	0.334	0.826	0.662
rs3739390	2.676	0.262	4.633	0.099
rs734701	0.674	0.714	1.829	0.401

SLE, systemic lupus erythematosus; HC, healthy control; SNP, single-nucleotide polymorphisms.

**Table 3:** Allele and genotype frequencies of six SNPs in the Ang2 gene in SLE patients and healthy controls.

SNPs	SLE [n (%)]	Controls [n (%)]	OR (95% CI)	*P* valu*e*
**rs12674822**				
TT	50 (21.3)	138 (36.3)	0.564 (0.358–0.890)	0.014
TG	124 (52.8)	147 (38.7)	1.314 (0.880–1.961)	0.182
TT+TG	174 (74.0)	285 (75.0)	0.951 (0.655–1.380)	0.791
GG	61 (26.0)	95 (25.0)	Reference	
T	224 (47.7)	423 (55.7)	0.725 (0.576–0.914)	0.006
G	246 (52.3)	337 (44.3)	Reference	
**rs1823375**				
GG	25 (10.6)	64 (16.8)	0.572 (0.339–0.964)	0.036
GC	100 (42.6)	155 (40.8)	0.944 (0.666–1.339)	0.748
GG+GC	125 (53.2)	219 (57.6)	0.835 (0.602–1.159)	0.281
CC	110 (46.8)	161 (42.4)	Reference	
G	150 (31.9)	283 (37.2)	0.790 (0.619–1.008)	0.058
C	320 (68.1)	477 (62.8)	Reference	
**rs1868554**				
TT	46 (19.6)	93 (24.5)	0.572 (0.364–0.898)	0.015
TA	99 (42.1)	183 (48.2)	0.625 (0.430–0.908)	0.014
TT+TA	145 (61.7)	276 (72.6)	0.607 (0.429–0.858)	0.005
AA	90 (38.3)	104 (27.4)	Reference	
T	191 (40.6)	369 (48.6)	0.725 (0.575–0.915)	0.007
A	279 (59.4)	391 (51.4)	Reference	
**rs2442598**				
GG	66 (28.1)	107 (28.2)	0.871 (0.563–1.348)	0.535
GA	101 (43.1)	177 (46.6)	0.806 (0.543–1.196)	0.284
GG+GA	167 (71.1)	284 (74.7)	0.830 (0.576–1.196)	0.317
AA	68 (28.9)	96 (25.3)	Reference	
G	233 (49.6)	391 (51.4)	0.928 (0.737–1.168)	0.523
A	237 (50.4)	369 (48.6)	Reference	
**rs3739390**				
GG	6 (2.6)	20 (5.3)	0.422 (0.166–1.071)	0.069
GC	35 (14.9)	87 (22.9)	0.566 (0.367–0.873)	0.010
GG+GC	41 (17.4)	107 (28.2)	0.539 (0.360–0.808)	0.003
CC	194 (82.6)	273 (71.8)	Reference	
G	47 (10.0)	127 (16.7)	0.554 (0.388–0.791)	0.001
C	423 (90.0)	633 (83.3)	Reference	
**rs734701**				
TT	40 (17.0)	90 (23.7)	0.580 (0.366–0.991)	0.020
TC	103 (43.8)	170 (44.7)	0.790 (0.548–1.139)	0.207
TT+TC	143 (60.9)	260 (68.4)	0.717 (0.511–1.008)	0.055
CC	92 (39.1)	120 (31.6)	Reference	
T	183 (38.9)	350 (46.1)	0.747 (0.591–0.944)	0.015
C	287 (61.1)	410 (53.9)	Reference	

SNP, single-nucleotide polymorphism; SLE, systemic lupus erythematosus; OR, odds ratio; 95% CI, 95% confidence interval.

### Association of Ang2 polymorphisms with clinical, laboratory features in SLE patients

Allele and genotypes frequencies of *Ang2* gene polymorphisms in SLE patients with different clinical features were summarized in Tables 4 and 5. Regarding to rs12674822, we found increased frequencies of TG and GG genotype in SLE patients with hypocomplementemia as compared to patients without this clinical feature ( P = 0.004). G allele frequency of rs12674822 was increased in patients with hypocomplementemia and proteinuria compared with patients without these features (P = 0.038; P = 0.043). Increased T allele frequency was observed in patients with thrombocytopenia when compared to patients without this specific features (P = 0.010) (Table 4). Significant differences for genotypes and allele frequencies of rs1823375 polymorphism was noted between patients with ds-DNA (+) and patients with ds-DNA (−) (P = 0.002; P = 0.030) (Table 4). When discussing rs1868554, frequencies of TT and TA genotypes were increased in patients with hematuria compared with patients without hematuria (P = 0.039). Frequency of T allele in patients with hematuria was increased in comparison with those without hematuria (P = 0.030) (Table 4). With respect to rs2442598, there was a higher frequency of G allele in patients with proteinuria as compared to those without proteinuria (P = 0.043) (Table 5). GC genotype frequency of rs3739390 was higher in patients with ds-DNA (+) and hematuria than that in patients without these features (P = 0.024; P = 0.011). Moreover, G allele frequency was increased in patients with hypocomplementemia, ds-DNA (+) and hematuria compared with those without these features (P = 0.049; P = 0.006; P = 0.003) (Table 5). For rs734701, frequency of genotypes and alleles in patients with all clinical features were comparable as compared to the patients without these features (all P > 0.050) (Table 5).

**Table 4:** Association between clinical, laboratory features and allele and frequencies of rs12674822, rs1823375, rs1868554 polymorphisms of Ang2 gene in SLE.

Clinical features	rs12674822							rs1823375							rs1868554						
	Genotype frequency (n)			*P* value	Allele frequency (n)		*P* value	Genotype frequency (n)			*P* value	Allele frequency (n)		*P* value	Genotype frequency (n)			*P* value	Allele frequency (n)		*P* value
	TT	TG	GG		T	G		GG	GC	CC		G	C		TT	TA	AA		T	A	
**Arthritis**																					
Positive	24	53	24	0.655	101	101	0.378	10	43	48	0.947	63	139	0.769	18	43	40	0.198	79	123	0.558
Negative	26	71	37		123	145		15	57	62		87	181		28	56	50		112	156	
**Rash**																					
Positive	19	54	23	0.673	92	100	0.926	8	43	45	0.606	59	133	0.647	20	38	38	0.798	78	114	0.996
Negative	31	70	38		132	146		17	57	65		91	187		26	61	52		113	165	
**Alopecia**																					
Positive	11	33	13	0.67	55	59	0.886	6	29	22	0.315	41	73	0.286	12	24	21	0.939	48	66	0.714
Negative	39	91	48		169	187		19	71	88		109	247		34	75	69		143	213	
**Fever**																					
Positive	8	26	8	0.393	42	42	0.636	3	20	19	0.709	26	58	0.835	10	17	15	0.744	37	47	0.483
Negative	42	98	53		182	204		22	80	91		124	262		36	82	75		154	232	
**Hypocomplementemia**																					
Positive	16	74	33	0.004	106	140	0.038	12	52	59	0.875	76	170	0.619	30	47	46	0.132	107	139	0.186
Negative	34	150	28		118	106		13	48	51		74	150		16	52	44		84	140	
**ds-DNA ( +)**																					
Positive	16	25	16	0.235	57	57	0.565	7	13	37	0.002	27	87	0.03	13	21	23	0.612	47	67	0.883
Negative	34	99	45		167	189		18	87	73		123	233		33	78	67		144	212	
**Thrombocytopenia**																					
Positive	13	13	5	0.01	39	23	0.01	3	13	15	0.999	19	43	0.818	6	14	11	0.926	26	36	0.823
Negative	37	111	56		185	223		22	87	95		131	277		40	85	79		165	243	
**Hematuria**																					
Positive	13	43	24	0.329	69	91	0.157	8	36	36	0.86	52	108	0.845	23	30	27	0.039	76	84	0.030
Negative	37	81	37		155	155		17	64	74		98	212		23	69	63		115	195	
**Proteinuria**																					
Positive	17	58	33	0.103	92	124	0.043	11	49	48	0.723	71	145	0.682	24	47	37	0.436	95	121	0.174
Negative	33	66	28		132	122		14	51	62		79	175		22	52	53		96	158	

SLE, systemic lupus erythematosus.

**Table 5:** Association between clinical, laboratory features and allele and frequencies of rs2442598, rs3739390, rs734701 SNPs of Ang2 gene in SLE.

Clinical features	rs2442598							rs3739390							rs734701						
	Genotype frequency (n)			*P* value	Allele frequency (n)		*P* value	Genotype frequency (n)			*P* value	Allele frequency (n)		*P* value	Genotype frequency (n)			*P* value	Allele frequency (n)		*P* value
	GG	GA	AA		G	A		GG	GC	CC		G	C		TT	TC	CC		T	C	
**Arthritis**																					
Positive	26	43	32	0.665	95	107	0.338	4	14	83	0.543	22	180	0.576	15	45	41	0.739	75	127	0.485
Negative	40	58	36		138	130		2	21	111		25	243		25	58	51		108	160	
**Rash**																					
Positive	25	38	33	0.311	88	104	0.178	5	15	76	0.103	25	167	0.07	18	39	39	0.685	75	117	0.963
Negative	41	63	35		145	133		1	20	118		22	256		22	64	53		108	170	
**Alopecia**																					
Positive	15	24	18	0.87	54	60	0.588	5	15	76	0.103	25	167	0.07	18	39	39	0.685	75	117	0.963
Negative	51	77	50		179	177		1	20	118		22	256		22	64	53		108	170	
**Fever**																					
Positive	11	19	12	0.938	41	43	0.877	1	5	36	0.854	7	77	0.574	8	17	17	0.87	33	51	0.942
Negative	55	82	56		192	194		5	30	158		40	346		32	86	75		150	236	
**Hypocomplementemia**																					
Positive	38	55	30	0.253	131	115	0.095	5	21	97	0.18	31	215	0.049	25	50	48	0.324	100	146	0.424
Negative	28	46	38		102	122		1	14	97		16	208		15	53	44		83	141	
**ds-DNA ( +)**																					
Positive	14	28	15	0.556	56	58	0.912	4	11	42	0.024	19	95	0.006	12	21	24	0.422	45	69	0.892
Negative	52	73	53		177	179		2	24	152		28	328		28	82	68		138	218	
**Thrombocytopenia**																					
Positive	9	10	12	0.342	28	34	0.456	1	4	26	0.826	6	56	0.928	5	14	12	0.984	24	38	0.969
Negative	57	91	56		205	203		5	31	168		41	367		35	89	80		159	249	
**Hematuria**																					
Positive	30	27	23	0.044	87	73	0.135	3	19	58	0.011	25	135	0.003	20	32	28	0.065	72	88	0.053
Negative	36	74	45		146	164		3	16	136		22	288		20	71	64		111	199	
**Proteinuria**																					
Positive	37	44	27	0.134	118	98	0.043	3	22	83	0.086	28	188	0.048	20	49	39	0.656	89	127	0.353
Negative	29	57	41		115	139		3	13	111		19	235		20	54	53		94	160	

SLE, systemic lupus erythematosus.

### Effect of Ang2 polymorphisms on its serum levels

**Figure 3:**
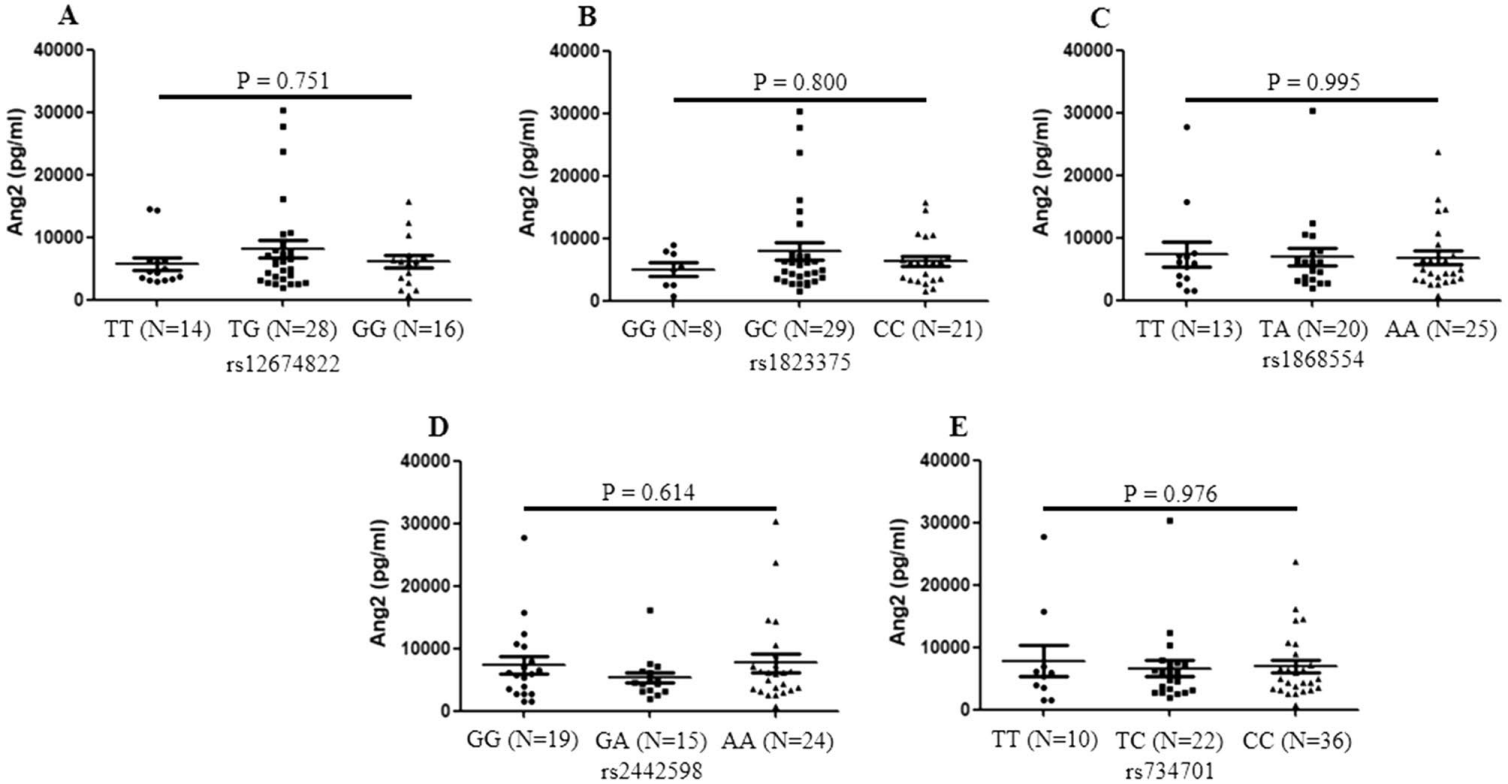
Association of Ang2 gene polymorphisms and serum Ang2 levels. Serum concentrations of Ang2 were tested by enzyme-linked immunosorbent assay and Ang2 polymorphisms (rs12674822, rs1823375, rs1868554, rs2442598 and rs734701) were genotyped by KASP method in SLE patients (n = 58). (**A**–**E**) Protein levels of Ang2 were not associated with genotypes of rs12674822, rs1823375, rs1868554, rs2442598 and rs734701 of Ang2 gene. Comparison of Ang2 values among three groups was conducted by the Kruskal–Wallis test.

Association between Ang2 polymorphisms and its serum levels was discussed in order to explore a possible impact of genetic variants on serum Ang2 levels in SLE patients. No significant difference was found in serum Ang2 levels among TT, TG, GG genotypes of rs12674822 in SLE patients (P = 0.751) (Fig. 3A). No significant difference in serum Ang2 levels among patients carrying GG, GC, CC of rs1823375 was observed (P = 0.800) (Fig. 3B). Patients carrying TT, TA, AA genotypes of rs1868554 presented comparable serum Ang2 concentrations as well (P = 0.995) (Fig. 3C). There was no significant association between serum Ang2 concentration and genotypes of rs2442598 and rs734701 (P = 0.614; P = 0.976) (Fig. 3D–E). Due to insufficient data for SLE cases carrying GG genotype of rs3739390, analysis was not conducted.

## Discussion

Previous study showed that in systemic sclerosis patients, serum Ang2 levels were increased compared with control group^21^. Similarly, higher circulating levels of Ang2 was found in knee osteoarthritis patients as compared to healthy subjects^5^. Plasma levels of Ang2 were increased in SLE patients when compared to healthy individuals, and were higher in cases with lupus nephritis (LN) than those without LN^1,20^. However, the sample sizes were small. In our two stage case–control studies, we used large sample sizes of SLE patients to discuss the association of serum levels of Ang2 in lupus. We found increased serum Ang2 levels in SLE patients in training cohort when compared with healthy controls, and in validation cohort when compared with other inflammatory autoimmune diseases. Our findings were similar to previous studies. In addition, we found that serum Ang2 levels were significantly related to clinical features of patients including rash and fever.

SLE is accompanied by different complications, such as infection, cardiovascular disorder, kidney disease and cancer^22^. Early diagnosis may be effective for patients. To date, diagnosis of SLE is partly based on different parameters like anti-double-stranded DNA (anti-dsDNA)^23,24^. In a previous study, sensitivity and specificity of anti-dsDNA were 52.4% and 94.2% for SLE diagnosis, respectively^25^. However, more biomarkers with high sensitivity and specificity will be helpful for diagnosis of lupus patients. In our study, we discussed ability of serum Ang2 as a biomarker for SLE. The AUC of Ang2 was 0.907 (95% CI: 0.856–0.958) in training cohort, which revealed a high potential to distinguish SLE patients from healthy subjects. In addition, we discussed the possibility of Ang2 to discriminate patients with SLE from other inflammatory autoimmune diseases by the validation cohort. Results suggested that serum Ang2 has potential to differentiate SLE patients from those with RA, OA. Compared with gout, SS and AS, serum Ang2 had good ability to distinguish SLE patients. To confirm serum Ang2 threshold of 2770.27 pg/ml as a marker for SLE, we examined the ability of Ang2 to distinguish SLE patients from non-SLE patients. Results showed a potential of serum Ang2 as a marker for SLE. However, to increase the power of our findings for serum Ang2 as a disease marker in SLE, there is a long way to go. For instance, more studies with multiple centers to confirm the potential of Ang2 as a marker when comparing serum Ang2 in SLE with different inflammatory diseases are needed. In addition, the performance of Ang2 in a specific cohort of lupus patients with pre-clinical, early and long-standing SLE disease, which are situations where Ang2 has demonstrated diagnostic or predictive utility is also needed discussion.

Angiogenesis is closely related to inflammation, in which neovascularization provides oxygen and nutrients for inflammatory tissues and promotes the transport of inflammatory cells^26,27^. The occurrence, location, extent and spread of inflammatory damage are mainly determined by activation of the endothelial layer^28^. Combination of Ang1 and Tie2 can maintain vascular integrity and homeostasis, inhibiting vascular leakage and inflammatory gene expression, and preventing leukocyte recruitment and migration^29–32^. However, as a key mediator of endothelial cell activation, Ang2 mediates endothelial cell inflammation and increases vascular inflammation by upregulating endothelial cell’s response to tumor necrosis factor (TNF)-α. The process results from destroying the protective signal of Ang1/Tie2^33^. In addition, Ang2 directly stimulates neutrophils, monocytes to adhere to and migrate to vascular disorders, leading to tissue infiltration of inflammatory cells^34^. These findings suggest that Ang2 mediates inflammatory process by activating endothelial cells. Angiogenesis and microvascular endothelial damage are involved in the pathogenesis of SLE, and endothelial dysfunction is one of the main causes of vascular injury in SLE^35,36^. Therefore, Ang2 may participate in the pathogenesis of SLE. However, the clear mechanism of Ang2 performed in needed to be discussed in the future.

In the present study, rs12674822 polymorphism in controls did not conform to HWE, which may relate to insufficient sample size and selecting control group without strict randomization. Thus, we need better design and larger sample size in future studies. Our findings about *Ang2* gene polymorphisms and SLE risk revealed that genotypes for rs12674822 (TT), rs1868554 (TT, TA and TT+TA) and rs734701 (TT) were negatively related to SLE susceptibility. Regarding to PV patients, both rs1868554 and rs3739390 showed no differences for genotypes distribution between cases and healthy individuals in a northern Chinese Han population^12^. With respect to rs1823375, frequency of GG genotype was reduced in SLE patients and was negatively related to risk of SLE in our findings. On the contrary, Dai et al. showed an increased frequency of GG genotype for rs1823375 in Taiwanese RA patients^13^. The inconsistence may relate to heterogeneity among different diseases, ethnicity and sample size. In addition, our data showed that *Ang2* gene polymorphisms were associated with certain clinical and laboratory characteristics. Patients carrying rs1868554 T allele and rs3739390 G allele were more likely to develop symptoms of hematuria. G allele of rs12674822 and rs2442598 was significantly associated with proteinuria in SLE patients. Significant correlation between rs12674822, rs3739390 G allele and hypocomplementemia in cases was observed. Moreover, the TT genotype for rs1868554 was related to hematuria in SLE patients. To our knowledge, the current study is the first to discuss relationship between *Ang2* gene polymorphisms and clinical, laboratory features in SLE patients. In addition, we analyzed association between Ang2 gene polymorphisms and serum Ang2 levels in SLE patients. Nevertheless, no significant results were found.

There are several limitations in our research. First, the rs12674822 in the control group do not conform to HWE. Thus, in future studies, determination of the healthy individuals should strictly follow the randomization principle. Second, the sample size is relatively limited and only includes patients from two hospitals. Therefore, studies with multi-center should be considered. Third, exploring interaction between gene polymorphisms and environment in our future study is necessary.

In summary, our data showed increased serum concentrations of Ang2 in SLE cases which may be a potential biomarker for this disorder, and Ang2 polymorphisms may correlate with SLE susceptibility in a Chinese Han population.

## Supplementary information

Supplementary Information 1.

Supplementary Information 1.

## Abbreviations

SLE: Systemic lupus erythematosus
Ang: Angiopoietin
Tie2: Tyrosine kinase receptor 2

## References

[1] Salama MK, Taha FM, Safwat M, Darweesh HE, Basel ME (2012). The Tie2 receptor antagonist angiopoietin-2 in systemic lupus erythematosus: its correlation with various disease activity parameters. Immunol. Invest..

[2] Barile-Fabris L, Hernández-Cabrera MF, Barragan-Garfias JA (2014). Vasculitis in systemic lupus erythematosus. Curr. Rheumatol. Rep..

[3] Lee WF (2019). Biomarkers associating endothelial Dysregulation in pediatric-onset systemic lupus erythematous. Pediatr. Rheumatol. Online. J..

[4] Kwon, Y.C., Chun, S., Kim, K., & Mak, A. Update on the genetics of systemic lupus erythematosus: genome-wide association studies and beyond. *Cells*. **8** (2019). 10.3390/cells8101180 PMC6829439 31575058

[5] Poonpet T (2018). Association between leukocyte telomere length and angiogenic cytokines in knee osteoarthritis. Int. J. Rheum. Dis..

[6] Guo Y, Xing E, Liang X, Song H, Dong W (2016). Effects of total saponins from Rhizoma Dioscoreae Nipponicae on expression of vascular endothelial growth factor and angiopoietin-2 and Tie-2 receptors in the synovium of rats with rheumatoid arthritis. J. Chin. Med. Assoc..

[7] Ziegler, T. et al. Angiopoietin 2 mediates microvascular and hemodynamic alterations in sepsis. *J. Clin. Inves*t. (2013). 10.1172/JCI66549 PMC3726157 23863629

[8] Kanakaraj P (2012). Simultaneous targeting of TNF and Ang2 with a novel bispecific antibody enhances efficacy in an in vivo model of arthritis. MAbs..

[9] Karampoor S, Zahednasab H, Ramagopalan S, Mehrpour M, Keyvani H (2016). Angiogenic factors are associated with multiple sclerosis. J. Neuroimmunol..

[10] Khattab, F.M., Said, N.M. Serum angiopoietin-2 level as a novel biomarker in atopic dermatitis. *Int. J. Dermatol.* (2019). 10.1111/ijd.14620 31682000

[11] Hu W (2019). Correlations between angiopoietin-2 gene polymorphisms and lung cancer progression in a Chinese Han population. J. Cancer..

[12] He L, Dang L, Zhou J, Bai J, Li YZ (2015). Association of angiopoietin-1, angiopoietin-2 and caspase-5 polymorphisms with psoriasis vulgaris. Clin. Exp. Dermatol..

[13] Dai C (2019). Correlation between genetic polymorphism of angiopoietin-2 gene and clinical aspects of rheumatoid arthritis. Int. J. Med. Sci..

[14] Petri M (2005). Review of classification criteria for systemic lupus erythematosus. Rheum. Dis. Clin. North. Am..

[15] Arnett FC (1988). The American Rheumatism Association 1987 revised criteria for the classification of rheumatoid arthritis. Arthritis. Rheum..

[16] Altman R (1986). Development of criteria for the classification and reporting of osteoarthritis. Classification of osteoarthritis of the knee. Diagnostic and Therapeutic Criteria Committee of the American Rheumatism Association. Arthritis. Rheum..

[17] Neogi T (2015). 2015 Gout classification criteria: an American College of Rheumatology/European League against rheumatism collaborative initiative. Arthritis. Rheumatol..

[18] Shiboski CH (2017). 2016 American College of Rheumatology/European league against rheumatism classification criteria for primary Sjögren's syndrome: a consensus and data-driven methodology involving three international patient cohorts. Ann. Rheum. Dis..

[19] van der Linden S, Valkenburg HA, Cats A (1984). Evaluation of diagnostic criteria for ankylosing spondylitis. A proposal for modification of the New York criteria. Arthritis. Rheum..

[20] El-Banawy HS, Gaber EW, Maharem DA, Matrawy KA (2012). Angiopoietin-2, endothelial dysfunction and renal involvement in patients with systemic lupus erythematosus. J. Nephrol..

[21] Takahashi T (2013). Dynamics of serum angiopoietin-2 levels correlate with efficacy of intravenous pulse cyclophosphamide therapy for interstitial lung disease associated with systemic sclerosis. Mod. Rheumatol..

[22] Gergianaki I, Bortoluzzi A, Bertsias G (2018). Update on the epidemiology, risk factors, and disease outcomes of systemic lupus erythematosus. Best. Pract. Res. Clin. Rheumatol..

[23] Aringer M (2019). 2019 European League Against Rheumatism/American College of Rheumatology classification criteria for systemic lupus erythematosus. Ann. Rheum. Dis..

[24] Qi S, Chen Q, Xu D, Xie N, Dai Y (2018). Clinical application of protein biomarkers in lupus erythematosus and lupus nephritis. Lupus.

[25] Bizzaro N, Villalta D, Giavarina D, Tozzoli R (2012). Are anti-nucleosome antibodies a better diagnostic marker than anti-dsDNA antibodies for systemic lupus erythematosus? A systematic review and a study of metanalysis. Autoimmun. Rev..

[26] Belmont HM, Abramson SB, Lie JT (1996). Pathology and pathogenesis of vascular injury in systemic lupus erythematosus. Interactions of inflammatory cells and activated endothelium. Arthritis. Rheum..

[27] Carmeliet P (2000). Mechanisms of angiogenesis and arteriogenesis. Nat. Med..

[28] Fiedler U, Augustin HG (2006). Angiopoietins: a link between angiogenesis and inflammation. Trends. Immunol..

[29] Kim I, Kim HG, So JN, Kim JH, Kwak HJ, Koh GY (2000). Angiopoietin-1 regulates endothelial cell survival through the phosphatidylinositol 3'-Kinase/Akt signal transduction pathway. Circ. Res..

[30] Thurston G (1999). Leakage-resistant blood vessels in mice transgenically overexpressing angiopoietin-1. Science.

[31] Papapetropoulos A (2000). Angiopoietin-1 inhibits endothelial cell apoptosis via the Akt/survivin pathway. J. Biol. Chem..

[32] Thurston G (2000). Angiopoietin-1 protects the adult vasculature against plasma leakage. Nat. Med..

[33] Fiedler U (2006). Angiopoietin-2 sensitizes endothelial cells to TNF-alpha and has a crucial role in the induction of inflammation. Nat. Med..

[34] Bezuidenhout L, Bracher M, Davison G, Zilla P, Davies N (2007). Ang-2 and PDGF-BB cooperatively stimulate human peripheral blood monocyte fibrinolysis. J. Leukoc. Biol..

[35] Clancy R (2001). Circulating activated endothelial cells in systemic lupus erythematosus: further evidence for diffuse vasculopathy. Arthritis. Rheum..

[36] Kluz J, Kopeć W, Jakobsche-Policht U, Adamiec R (2009). Circulating endothelial cells, endothelial apoptosis and soluble markers of endothelial dysfunction in patients with systemic lupus erythematosus-related vasculitis. Int. Angiol..

