# Supplementary material for: Serum levels and gene polymorphisms of angiopoietin 2 in systemic lupus erythematosus patients
Source: Sci Rep. 2021 Jan 8;11:10. doi: 10.1038/s41598-020-79544-z (PMC7794606; doi:10.1038/s41598-020-79544-z)
Supplement: Supplementary file 2 — Supplementary Information 1. [file 41598_2020_79544_MOESM2_ESM.doc]

| Supplementary table 1 Primer sequence of six SNPs | | | |
| --- | --- | --- | --- |
| SNPs | Primer_AlleleFAM | Primer_AlleleHEX | Primer_Common |
| rs12674822 | CACTTGTCTGGCCCAACCCTG | CACTTGTCTGGCCCAACCCTT | CTCACCTTTTCTGGGCCTCAAATAATATA |
| rs1823375 | GGTCAGGGCAGGTGAAGGG | GGTCAGGGCAGGTGAAGGC | GTGACTTCTCTTAGGGAGCACACTT |
| rs1868554 | ACTGTAAATTACCCAGGCCAGGTAT | ACTGTAAATTACCCAGGCCAGGTAA | GGTGGCTGGAGGTCTCATATCTTAA |
| rs2442598 | GTGTGCGAGGACAGTGTGTGTTT | GTGCGAGGACAGTGTGTGTTC | TGTGTCAACCATCAAGAAGAGGACAAAAT |
| rs3739390 | GGACGTGTGTTTGCCCTCAAG | GGACGTGTGTTTGCCCTCAAC | CTTCAGTAATAAACCAGCAGCTTAGCAAA |
| rs734701 | AGATAAGAGAATAGAATAACAAATTACTTGAC | CAGATAAGAGAATAGAATAACAAATTACTTGAT | TGTTGTGATATTGTGGAAAGACCTGGTA |

SNP, single nucleotide polymorphism.
